# Supplementary material for: Identification of locally activated spindle-associated proteins in oocytes uncovers a phosphatase-driven mechanism
Source: J Cell Sci. 2025 Nov 20;138(22):jcs264161. doi: 10.1242/jcs.264161 (PMC12669969; doi:10.1242/jcs.264161)
Supplement: Supplementary information [file joces-138-264161-s1.pdf]

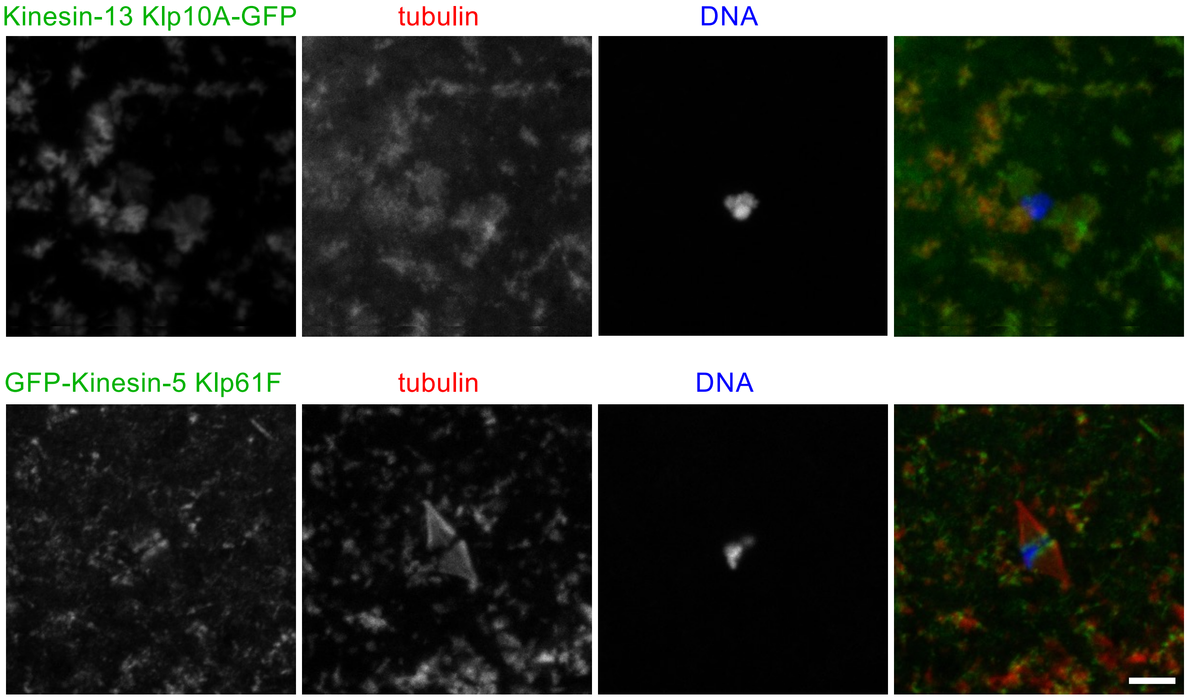

Fig. S1. Spindle proteins that are not regulated spatially in oocytes in terms of microtubule binding

Mature oocytes expressing a GFP-tagged protein were incubated with taxol and immunostained using GFP and α-tubulin antibodies. GFP-tagged Kinesin-13 Klp10A and Kinesin-5 Klp61F localised to both spindle and ectopic microtubules with similar intensities. A total of 10 and 23 oocytes were examined for each. Bar=5 μm.

Journal of Cell Science • Supplementary information

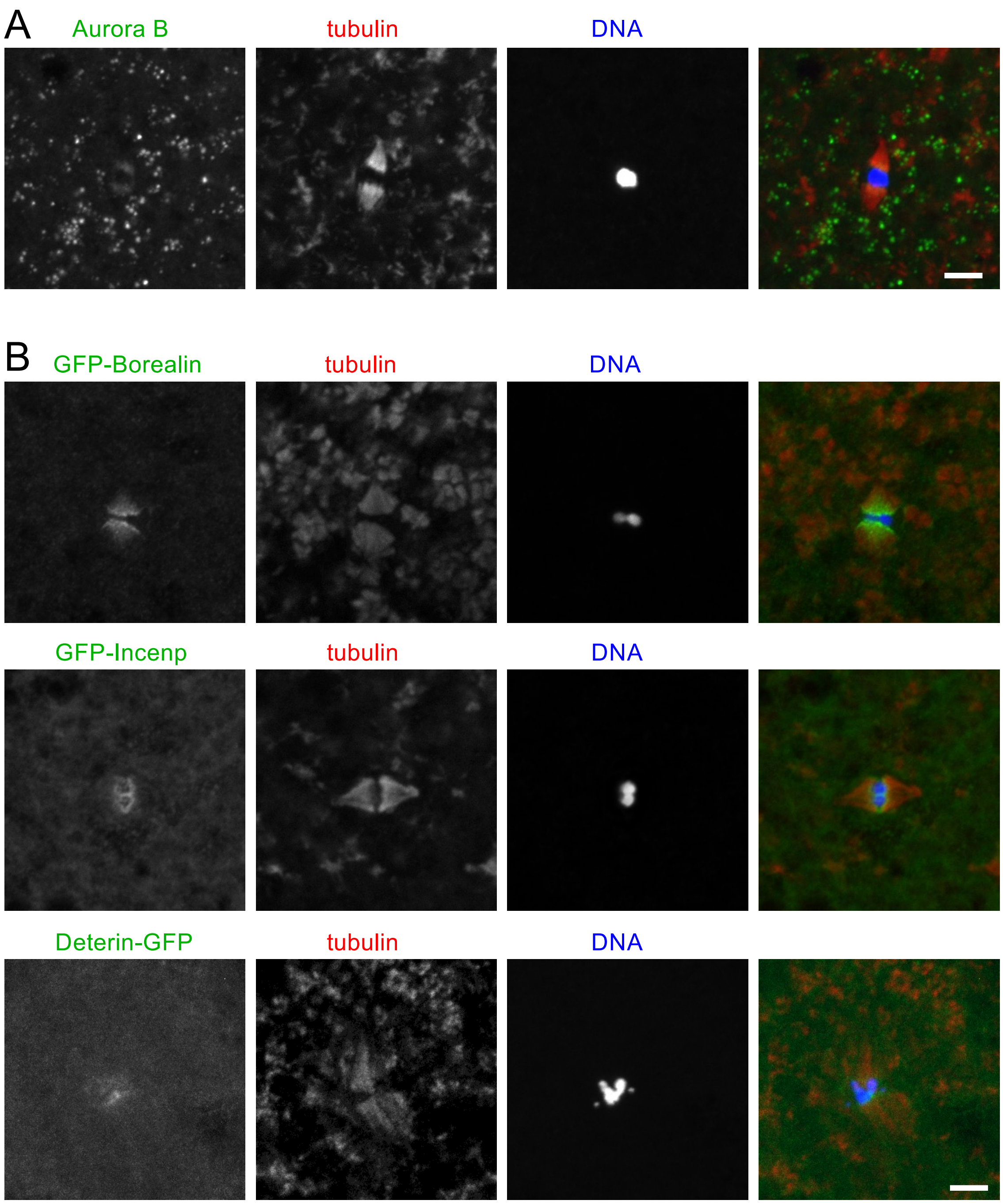

Fig. S2. Microtubule binding of the CPC subunits are spatially regulated in oocytes

(A) Wild-type mature oocytes arrested in metaphase I were incubated with taxol and immunostained using Aurora B and α-tubulin antibodies. Aurora B was concentrated on spindle microtubules near chromosomes, not on ectopic microtubule clusters. A total of 23 oocytes were examined. Bar=5 μm.

(B) Mature oocytes expressing a GFP-tagged protein were incubated with taxol and immunostained using GFP and α-tubulin antibodies. GFP-tagged Borealin, Incenp and Deterin were concentrated on spindle microtubules near the chromosomes, not on ectopic microtubule clusters. A total of 19, 16 and 14 oocytes were examined for each. Bars=5 μm.

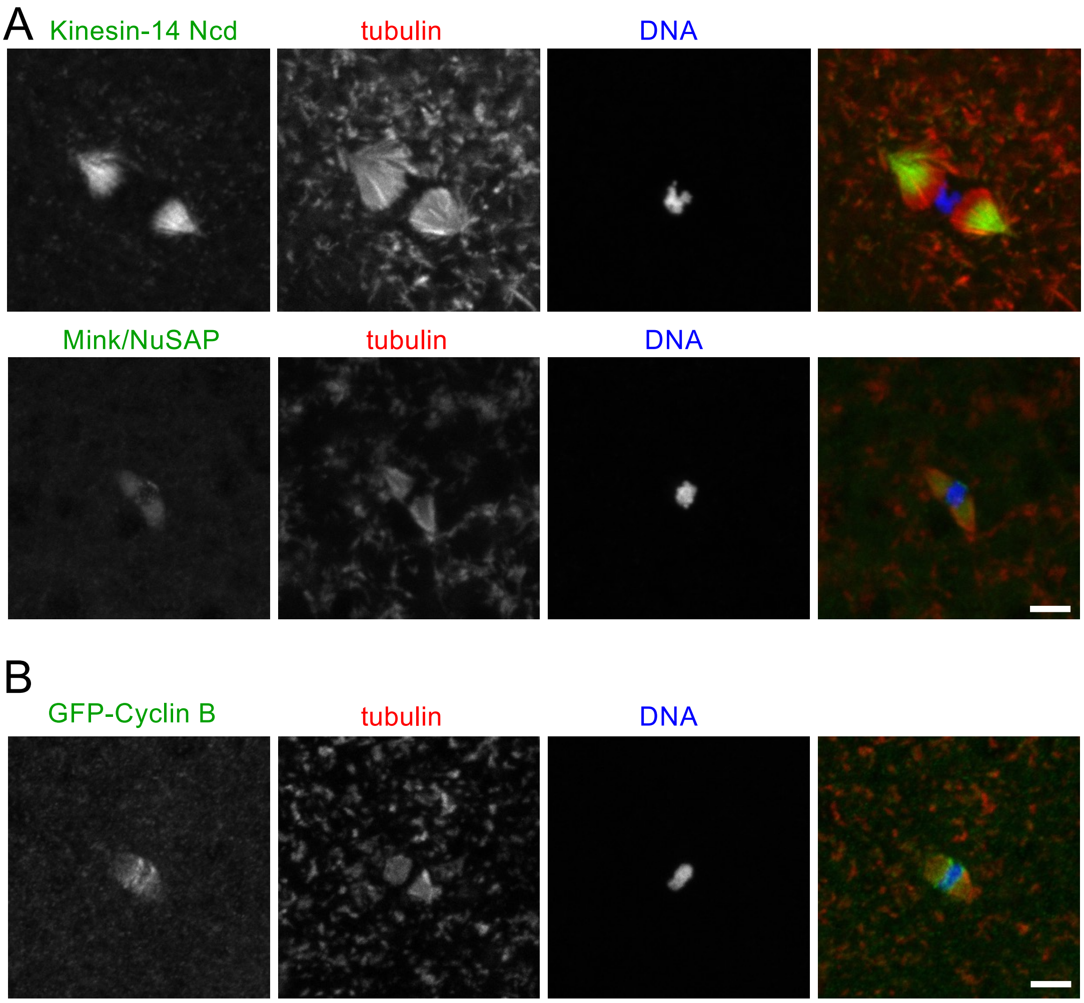

Fig. S3. Microtubule binding of Ncd, Mink and Cyclin B is spatially regulated in oocytes

(A) Wild-type mature oocytes arrested in metaphase I were incubated with taxol and immunostained using antibodies against Ncd or Mink and α-tubulin. Ncd and Mink were concentrated on spindle microtubules, but much less on ectopic microtubule clusters. A total of 85 and 42 oocytes were examined for each. Bar=5 μm.

(B) Mature oocytes expressing a GFP-tagged Cyclin B were incubated with taxol and immunostained using antibodies against GFP and α-tubulin. GFP-tagged Cyclin B was concentrated on spindle microtubules near chromosomes, not on ectopic microtubule clusters. A total of 25 oocytes were examined. Bar=5 μm.

Journal of Cell Science • Supplementary information

A

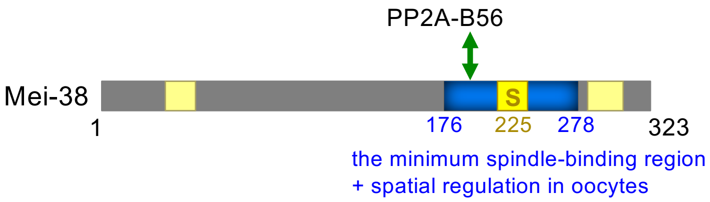

B

```
                conserved region 1
MDNASSTFGQ SDFDWDAINV GDYQLDHSQN FFARANKENN RSLSNVLLCT TPQKIGREQR  60

DGDFDGKLIS AGDLKKESPK DTTEASKDAL TIKPKVEPHD VPELKLKTEL QELAQVSPLD 120
                                   | 95–

NGPEPQDDIK QIVPLNWGKD GDDDEKHMTE ADQHLVHQEA RPPGVGAKLR LEWSPPRHRE 180
                        | 143–                                    | 176–
                                                  conserved region 2
HEGAGASATS AAPKAYQFKD VYESKRQQAM RKRSEEESKA RQFHSRPMPN FKAMHKRMNE 240
                  | 195–                                 225
                                                        conserved region 3
LAVIHKITVP RTPETLKHSQ SNVERRRDDD PADQARLHPA GTRSRPFHLR SEQRVRDRRE 300
–242 |                                   –278 |

FDAAVQVSLE QKKKEVKTKS SSP 323
```

XXXXX: conserved regions among animals and plants defined by Goshima (2011)

S: highly conserved serine at 225

XX: PP2A-B56 docking motif

| nnn– : the start of a truncated Mei-38

–nnn | : the end of a truncated Mei-38

| 176–242 | : the minimum microtubule-binding region

| 176–278 | : the minimum spindle-binding region + spatial regulation in oocytes

Fig. S4. The Mei-38 amino acid sequence

(A) Schematic diagram of the Mei-38 primary structure.

(B) The Mei-38 amino acid sequence based on the sequence of the cDNA pAG41. Key residues are marked.

Journal of Cell Science • Supplementary information

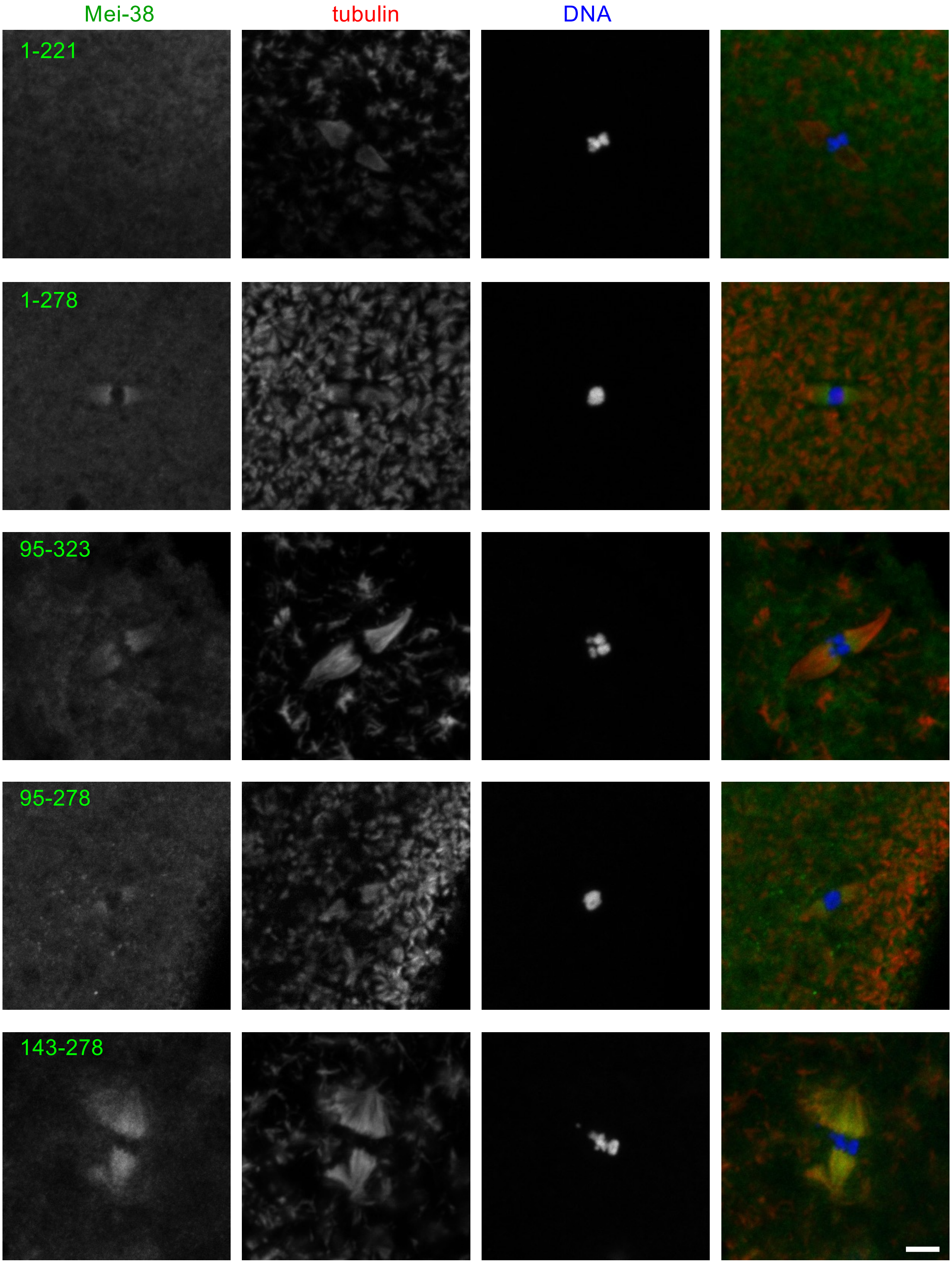

Fig. S5. Localisation of various Mei-38 truncations in taxol-treated oocytes After taxol treatment, mature oocytes expressing various GFP-tagged Mei-38 truncations were immunostained using GFP and α-tubulin antibodies. Quantification of these truncations and the images and quantification of other truncations are shown in Fig.2. Bar=5 μm.

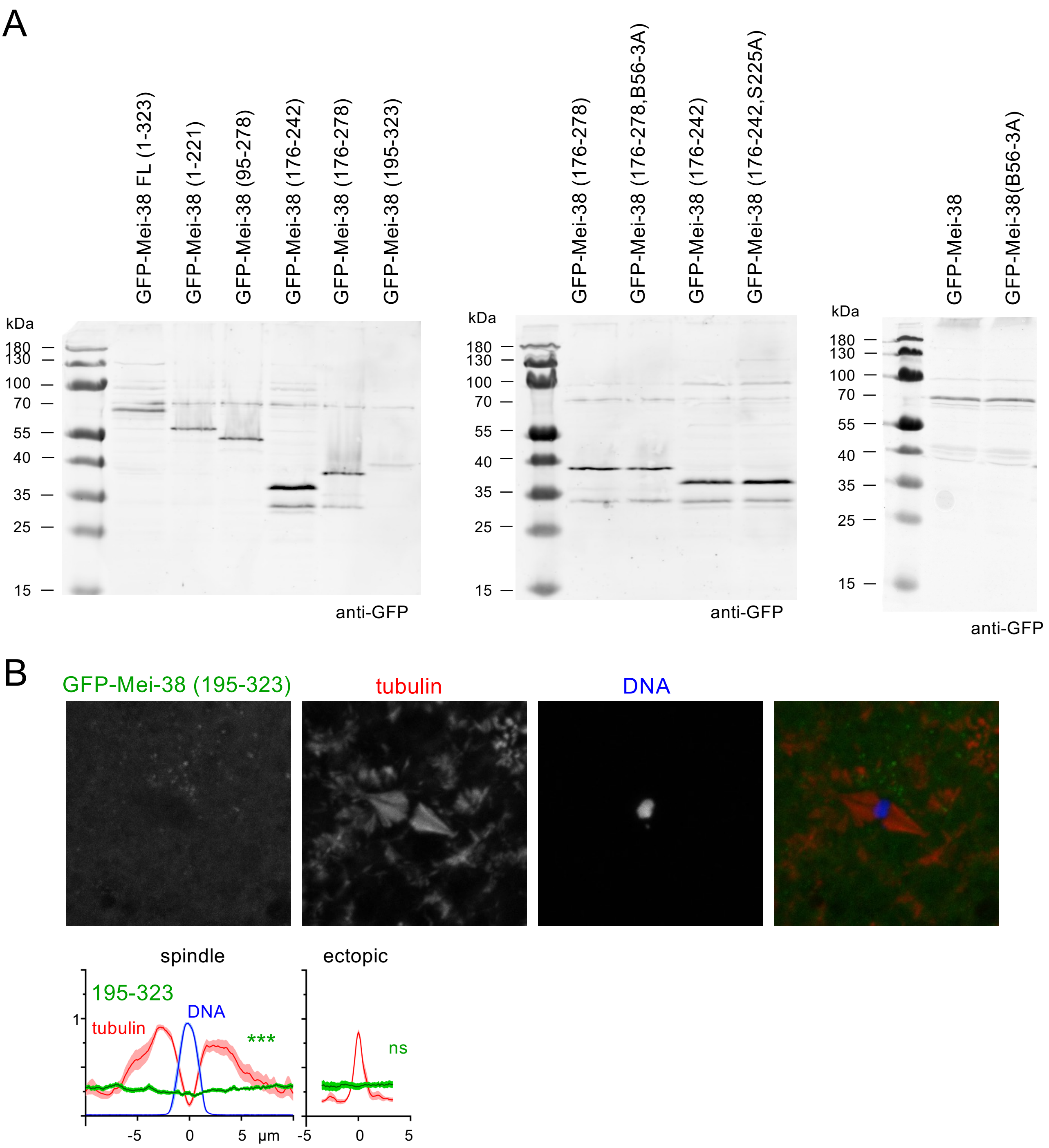

Fig. S6. Western blots of various GFP-Mei-38 truncations and mutations

(A) Ovaries expressing various forms of GFP-Mei-38 were run on SDS-PAGE, and western-blotted using an anti-GFP antibody to estimate the size and quantity of GFP-tagged proteins. Comparable amounts of GFP-tagged proteins were produced except Mei-38(195-323). The experiment was done once.

(B) Immunostaining of GFP-tagged Mei-38(195-323) in taxol-treated oocytes. Note that the amount of this truncated protein in ovaries was much less than that of the full-length Mei-38 or the other truncated proteins. Quantification was carried out as Fig.2B. *** and ns indicate $p<0.001$ and $p>0.05$, respectively, in two-tailed t-tests when the signal intensity of a GFP-Mei-38(195-323) is compared to that of GFP-Mei-38. n=9, $p=2.4x10^{-11}$ (spindle), 0.74 (ectopic). The spreads of the individual data points used for statistical analysis are shown in Fig. S10.

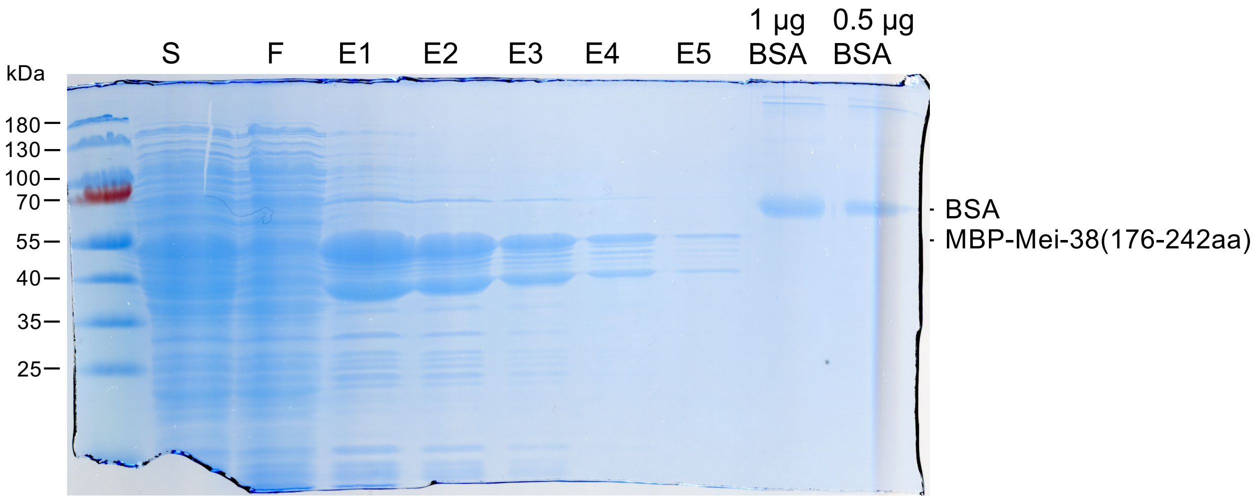

Fig. S7. Purification of MBP-Mei-38(176-242)

After bacterial cells expressing MBP-Mei-38(176-242) were lysed, the lysate was centrifuged and the supernatant (S) was run through an amylose column. The flowthrough fraction (F) and elution (E1-E5) using buffer containing maltose were analysed together with BSA for quantification.

Journal of Cell Science • Supplementary information

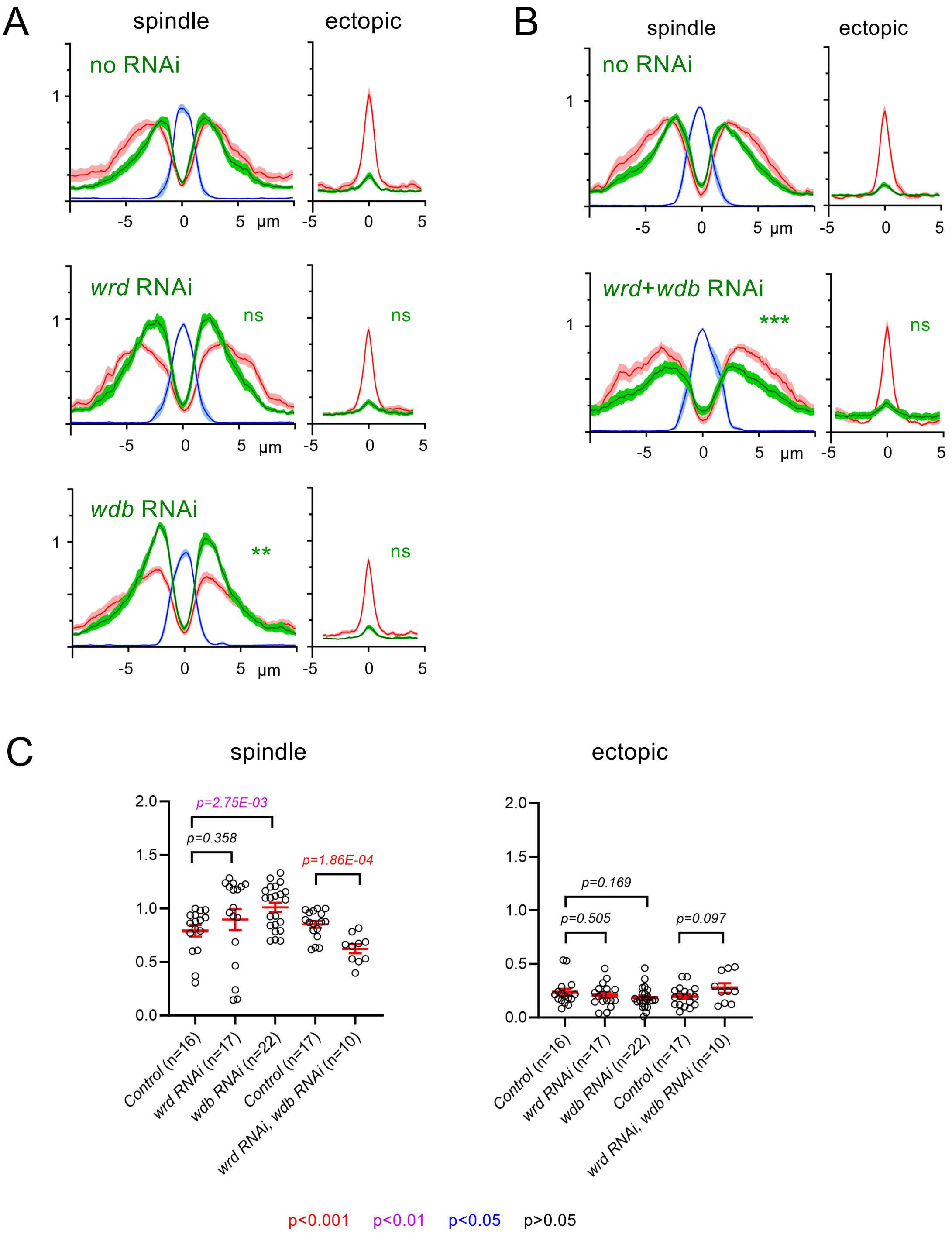

Fig. S8. Localisation of Mei-38 in taxol-treated oocytes with RNAi against PP2A-B56
Localisation of GFP-Mei-38 in taxol-treated oocytes expressing shRNAs against one or both of the PP2A-B56 isoforms. The signal distribution was quantified as **Fig.2B**. ***, ** and ns indicate p<0.001, p<0.01 and p>0.05, respectively, in two-tailed t-tests when the signal intensity of a GFP-Mei-38 in RNAi is compared to no RNAi. (A) Single RNAi against *wrd* or *wdb.* n=16, 17, 22 for no RNAi, *wrd* RNAi, *wdb* RNAi, respectively.
p=0.36, 0.0028 (spindle) and p=0.51, 0.17 (ectopic) for *wrd* or *wdb* RNAi, respectively.(B) Double RNAi against *wrd* and *wdb*. n=17, 10 for no RNAi, *wrd*+*wdb* double RNAi.
p=0.00019 (spindle) and p=0.097 (ectopic). (C) The spreads of the data points are shown with the means and standard errors as lines and whiskers.

Journal of Cell Science • Supplementary information

A (Figure 3B)

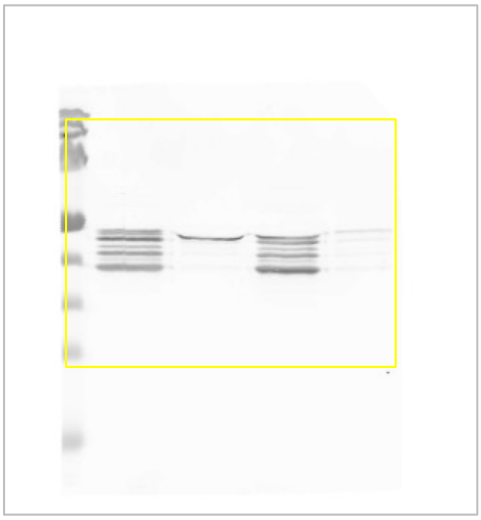

B (Figure S6)

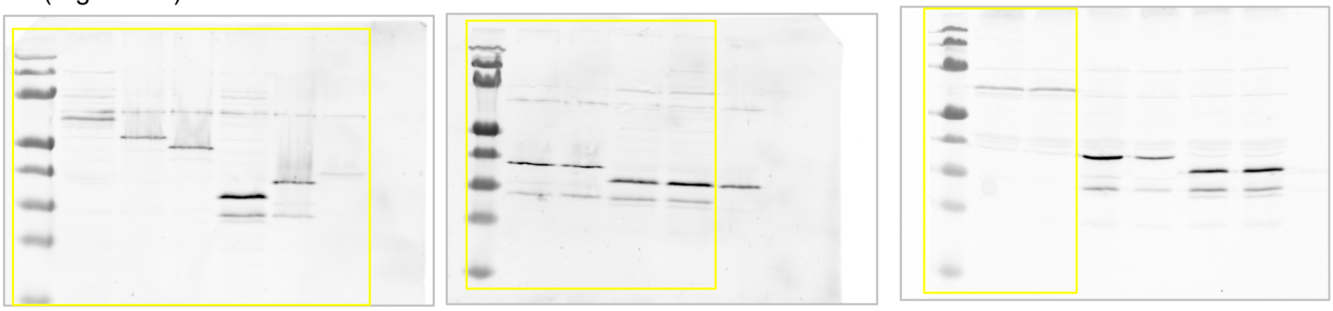

Fig. S9. Blot transparency

(A) The full, uncropped blot of Figure 3B. The cropped area is shown as a box.

(B) The full, uncropped blots of Figure S6. The cropped areas are shown as boxes. Lanes that were cropped out represent other fragments not included in our final analysis or duplicated samples that we did not use for immunostaining.

Journal of Cell Science • Supplementary information

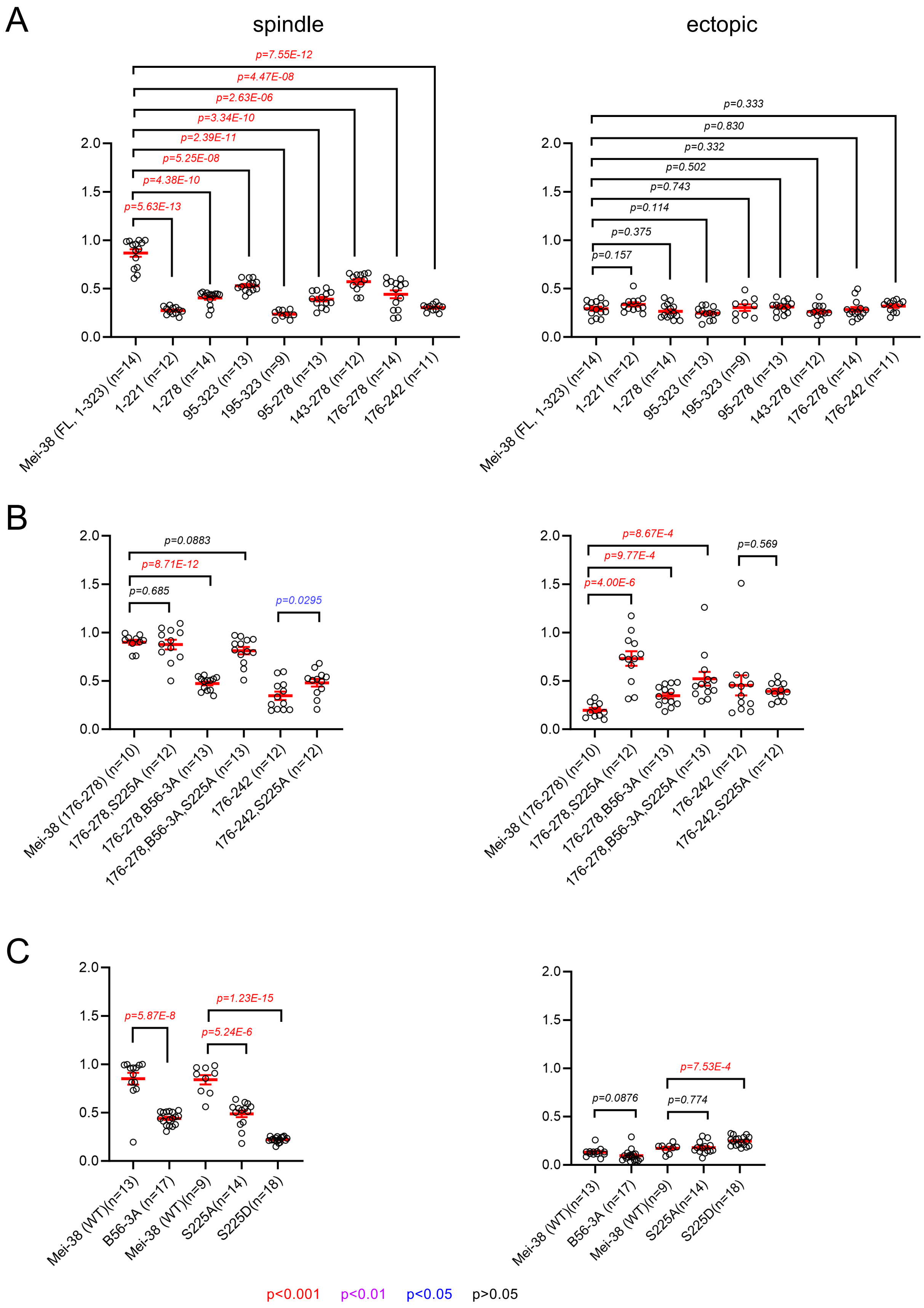

Fig. S10. The spreads of the individual data points used for statistical analysis

The spreads of the data points are shown with the means and standard errors as lines and whiskers. The statistics are presented in (A) Fig. 2, (B) Fig. 4, 5, S6, (C) Fig. 6.

Journal of Cell Science • Supplementary information

Table S1. Measurements of the signal intensities

An Excel file containing all measurements of signal intensities used for producing graphs in the figures except Fig. S8.

Available for download at
https://journals.biologists.com/jcs/article-lookup/doi/10.1242/jcs.264161#supplementary-data

Table S2. Measurements of the signal intensities in Fig. S8.

An Excel file containing all measurements of signal intensities used for producing graphs in Fig. S8.

Available for download at
https://journals.biologists.com/jcs/article-lookup/doi/10.1242/jcs.264161#supplementary-data
